# Finding counterfeited banknotes: the roles of vision and touch

**DOI:** 10.1186/s41235-020-00236-3

**Published:** 2020-08-20

**Authors:** Frank van der Horst, Joshua Snell, Jan Theeuwes

**Affiliations:** 1 De Nederlandsche Bank (DNB), Spaklerweg 4, 1096 BA Amsterdam, The Netherlands; 2 Department of Experimental and Applied Psychology, Vrije Universiteit, Amsterdam, The Netherlands; 3 Institute of Brain and Behavior Amsterdam (iBBA), Amsterdam, The Netherlands

**Keywords:** Attention, Decision-making, Gist, Vision, Touch, Authentication, Banknotes, Counterfeits

## Abstract

Central banks incorporate various security features in their banknotes to enable themselves, the general public, retailers and professional cash handlers to detect counterfeits. In two field experiments, we tested central bank counterfeit experts and non-experts (the general public) in their ability to detect counterfeited euro banknotes. We varied exposure duration and perceptual modality (sight, touch or both). The counterfeit banknotes were actual counterfeits taken out of circulation. Experiment 1, in which participants only viewed the banknotes, showed that experts did reasonably well in detecting counterfeits even when exposure duration was limited to 500 ms. Non-experts did not reach the criterion for decent performance, marked by *d’* = 1.25, although they did perform above chance. In Experiment 2, participants could both see and touch the banknotes, which resulted in better performance especially with longer exposure durations. The main finding of the current study is that visual information mostly impacts the decision-making process during the first glance, whereas tactile information increasingly aids performance as it continues to be accrued over time. Implications for the design of security features of new banknotes are discussed.

## Significance statement

The present study investigated how well experts (bank employees dealing with counterfeiting) and non-experts (the general public) are able to detect counterfeited banknotes. The results show that the general public is able to do this well above chance even when they see the banknote for only 500 ms. Experts performed much better. When non-experts and experts can both see and touch the banknote performance becomes much better, especially when there is ample time to check these banknotes. It is recommended that when central bankers design new banknotes, they should continue to consider security features that appeal to both touch and vision.

## Introduction

In 2016, consumers in the euro area made on average 1.2 cash payments per day (Esselink & Hernandez, 2017). These cash transactions were largely habitual (Van der Horst & Matthijsen, 2013). Upon receiving a banknote – either from a retailer or in a person-to-person transaction – people typically prioritize determining its value. Determining whether the banknote is fake or real is regarded as less important (Klöne, Vrakking, & Zondervan, 2019). Research has shown that Dutch citizens have strong confidence in the authenticity of euro banknotes because the likelihood of receiving a counterfeit is very low (Van der Horst, De Heij, Miedema, & Van der Woude, 2017). For example, in Europe in 2018, the number of counterfeit euro banknotes that were removed from circulation (563,000) constituted only 0.003% of the number of genuine euro banknotes in circulation (22 billion) (European Central Bank (ECB), 2019). Mainly because of this, people tend to not authenticate banknotes, especially when, at first glance, the banknote appears normal (Van der Horst et al., 2017). Indeed, in the study of Klöne et al. (2019), 70% of a sample of Dutch respondents claimed to have never intentionally and consciously authenticated a banknote in the last 5 years.

**Fig. 1:**
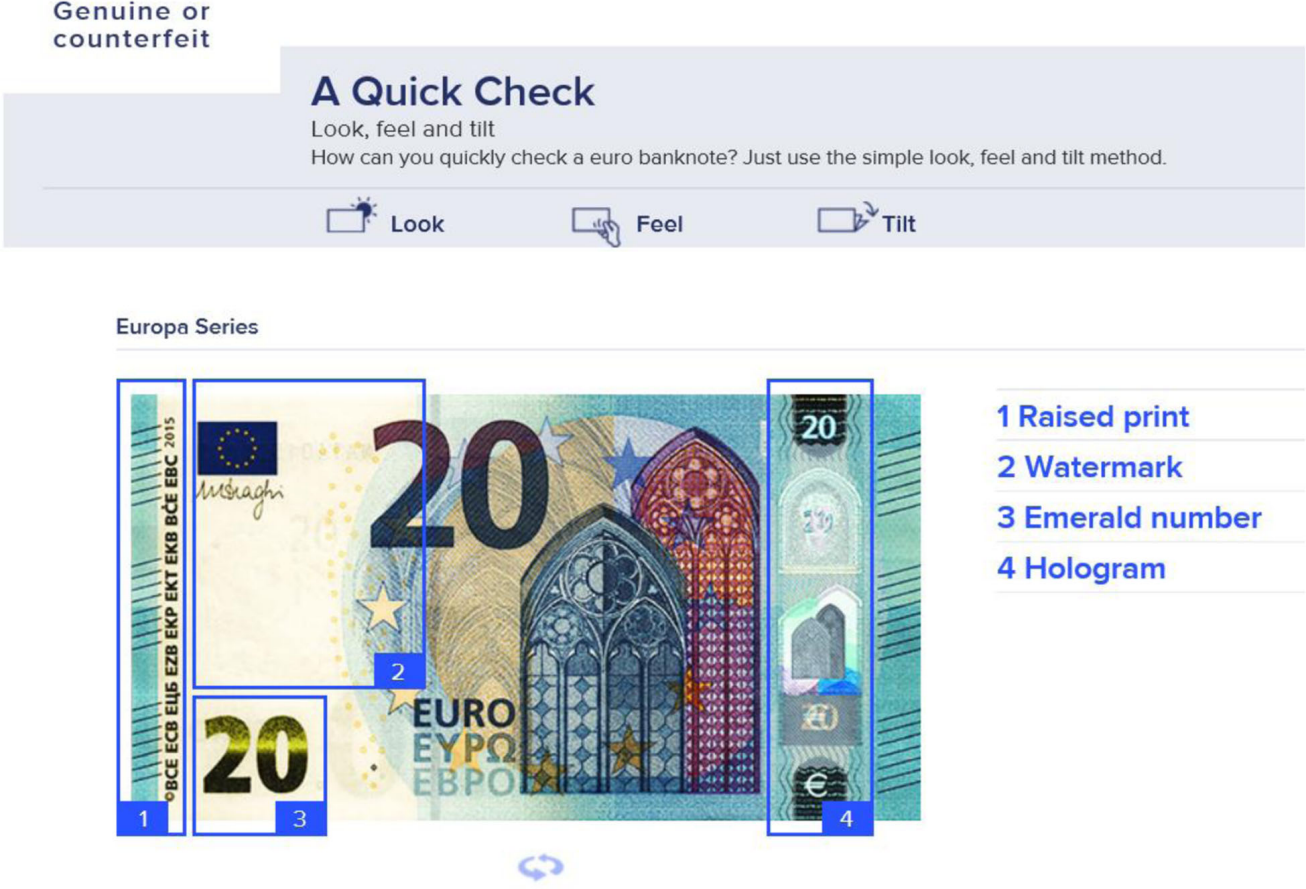
Security features for the public shown on the De Nederlandsche Bank (DNB) website (www.dnb.nl/echtofvals)

The relatively high levels of trust exhibited by the general public fuel the need for banknotes of which the authenticity can be easily confirmed – which, in consequence, should boost one’s ability to detect deviants beyond the limits imposed by naïveté. For these reasons, all central banks incorporate various security features in their banknotes to assist various user groups in identifying counterfeits without specialized equipment. Examples are a watermark, a security thread that is imbedded in the paper, optically changing elements, security foils (sometimes including holograms), paper structure and *alto-relievo* induced by *intaglio* printing (raised ink). These authentication features appeal to two of our five senses, namely sight and touch (see Fig. 1).

As of yet, we do not have complete knowledge of the factors contributing to counterfeit detectability. In particular, we know little of the respective contribution of visual and haptic perception in the decision-making process. We additionally do not know how much time is needed to ensure that the exploitation of these senses prompts at least a decent detection performance. For instance, would one either feel anomalies within a split second or never at all, or would anomaly detection improve as one accrues more haptic evidence over time? Additionally, we do not yet know how these factors are influenced by expertise. Specifically, might expertise increase the value of evidence accrued beyond the first impression? The present study is aimed at answering these questions.

### Two decision-making systems

Before reporting our experiments, we should outline a few theoretical constraints. Prior research has led one to believe that humans have two separate cognitive systems driving decision-making. One of these is fast, automatic and largely non-conscious; a type of processing that has been labeled System-1 or Type-1 processing (Frankish, 2010; Kahneman, 2010). In the present context it could be argued that a typical cash transaction would solely involve Type-1 processing. However, in probing counterfeit detection with a cognitive experiment, we may inherently be unable to assess Type-1 processing: specifically, asking participants whether a given banknote is real or fake is likely to induce atypical levels of distrust. In consequence, authentication would consist of a slower, controlled and conscious decision-making process, which in the literature has been labeled Type-2 processing (Frankish, 2010; Kahneman, 2010). See Klöne et al. (2019) for a discussion of factors driving a more deliberate banknote verification process.

Hence, in assessing the interactions of perceptual modality, time and expertise in the detection of counterfeit banknotes, our conclusions will largely pertain to Type-2 decision-making processes. The extent to which findings may inform us about Type-1 processes will be addressed in the “General discussion” section.

It must be stressed that human authentication has its limits even when invoking Type-2 processes. For example, in an experiment in which genuine and manipulated photographs were presented on a computer screen, it was shown that people have poor ability in identifying whether an image is the original or has been manipulated (Nightingale, Wade, & Watson, 2017). It has been argued that our inability to detect changes is largely driven by the fact that the overall gist of the percept remains unaltered (e.g., Standing, Conezio, & Haber, 1970).

In addition to limitations in perception, we must highlight the “prevalence effect.” Visual search experiments are perceptual tasks that require active scanning of the visual environment for a particular object or feature (the target) among other objects or features (the distractors). In most visual search experiments, targets appear on at least 50% of trials (Wolfe & van Wert, 2010). However, Wolfe and van Wert showed that when targets were rare (1% prevalence) observers made more than four times the number of “miss” errors made when targets were common (50% prevalence). To miss a disproportionate number of targets when these targets are rare, is especially problematic in important everyday contexts such as medical or airport screening (Wolfe & Van Wert, 2010). Wolfe, Horowitz, and Kenner (2005) showed that if observers repeatedly do not see their target, they will more probably fail to notice it once it does appear. Undoubtedly this prevalence effect also impacts on counterfeit detection as well, given that counterfeits in everyday life are extremely rare (Rich et al., 2008).

### About time

The act of accepting a banknote is performed rapidly. An internal DNB cashier field study (Zondervan, Heinen, & Heuvel, 2019) shows that most cashiers make – implicitly or explicitly and without the use of authenticating devices – the decision of whether or not to accept a banknote within 3 s. According to Layne-Farrar (2011) it takes only 1–2 s for waiters to pick up money from a table for a tip and pocket it. The simple task of accepting a banknote and storing it in your wallet is probably within that range of time. Presumably, this is also the time that the banknote has been authenticated, at least implicitly. Some national central banks of the Eurosystem (e.g., Bank of Italy (2020) and Bank of Finland (2020)) state on their websites that it only takes a few seconds to (explicitly) authenticate a banknote. However, as far as is known there has been no empirical evidence regarding the speed with which banknote can be recognized as counterfeit or genuine. In the current study, the task was to decide whether a banknote was counterfeit or genuine and the exposure duration to the banknote was systematically varied.

As noted before, it is unknown whether counterfeit detection would benefit from a longer exposure duration. However, research on scene perception may be somewhat informative, as it has revealed that people are able to recognize complex, real-world scenes at a mere glance, regardless of the visual complexity of the scene (see, for instance, Fei-Fei, Lyer, Koch, & Perona, 2007; Oliva, 2005). On the other hand, it should be noted that this type of recognition concerned the gist of the scene (e.g., “it is an outdoor scene with mountains”) without concern for specific features or details. Possibly, more fine-grained perception is a prerequisite for successful counterfeit detection.

It is generally believed that presenting a display for only 200 ms should be enough for detecting basic features. Because the time it takes to make a saccadic movement is at least 200 ms, such a task is completed in a single glance (Healey & Enns, 2012). The recognition and discrimination of patterns appears to take longer. According to Fei-Fei et al. (2007) observers need a presentation time of 500 ms to be able to almost perfectly categorize outdoor and indoor scenes. Furthermore, a study by Greene, Botros, Beck, and Fei-Fei (2015) showed that participants can make an adequate description of typical real-world situations scenes after 506 ms, although it takes participants longer to understand and even perceive improbable visual images (e.g., a press conference being convened under water), indicating that our rapid scene-categorization abilities depend critically on our prior experience with real-world environments (Greene et al., 2015).

As noted earlier, one of the goals of this study was to determine the lower limit on how rapidly people can distinguish counterfeits from real banknotes. If counterfeits are distinct from genuine banknotes by virtue of features that stand out (e.g., Theeuwes, 1992) then one should be able to do this very rapidly. In the set of exposure times employed in our experiments, we therefore incorporated a 500-ms condition, which represents a time in which one or two eye-movements can be made. We also tested longer exposure durations of 1000 ms and (up to) 10 s to determine whether a longer exposure duration would improve performance. Indeed, if the detection of counterfeit banknotes requires the processing of specific details, we would expect that a longer exposure duration would greatly improve performance. As an upper limit we used an exposure duration of 10 s, as it was previously shown that the hit rate in detecting a counterfeit does not increase beyond an exposure duration of 10 s (Van der Horst, Eschelbach, Sieber, & Miedema, 2016).

As argued before, it remains to be seen to what extent these temporal constraints are modulated by certain factors, such as expertise and perceptual modality. Whereas the above findings pertain to vision, haptic perception is also likely to play a considerable role in counterfeit detectability. Below, we provide a review of the tenets of touch with respect to counterfeit detectability.

### Touching on touch

When both visual and haptic perception are available, it is likely that they will play an interactive role (Wijntjes, 2009, cited by De De Heij, 2017; Kandula, Hofman, & Dijkerman, 2015). An example of such an interaction is the rubber-hand illusion: watching a rubber hand being stroked, while one’s own unseen hand is synchronously stroked, may cause the rubber hand to be attributed to one’s own body (Tsakiris & Haggard, 2005).

The conception that haptic perception is likely to play a considerable role is further fueled by the fact that it may simply contribute a good deal of new information. Consider, for instance, that when using a banknote, people might see only one side of it, but will always feel both sides.

Haptic perception typically involves active manual exploration. In general, when exploring objects haptically, people tend to rely on their experiences with the external world of surfaces and object properties such as roughness, shape, weight, material characteristics, contour, etc. According to Lederman and Klatzky (1987), there are basically six types of haptic exploration: (1) lateral motion, typically used to explore textures; (2) pressure, to determine hardness; (3) static contact, to assess temperature; (4) unsupported holding, for judging weight; (5) enclosure for estimating size; and (6) contour tracking, to determine the shape. With respect to assessing counterfeit banknotes haptically, we hypothesize that lateral motion (exploring texture) and pressure (hardness of the surface) are the most important types of haptic exploration. Note, however, that we do not manipulate or control for the type of haptic exploration used by participants in the present study. Hence, if we are to establish a considerable role for haptic perception, we would not be able to make claims about specific strategies; (and, analogously, we will not be able to make claims about specific strategies in the visual domain).

**Fig. 2:**
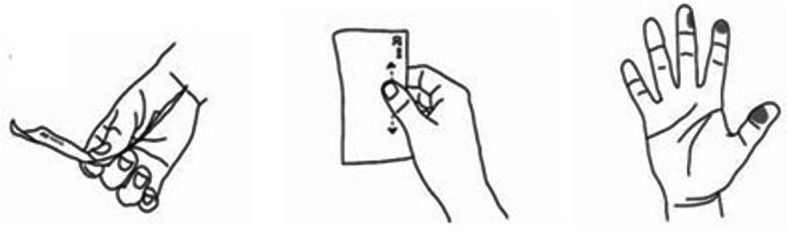
Haptic banknote interaction. Left: movement over the surface. Bending of the paper, fingers on two sides. Thumb on the front and index finger on reverse. The middle finger is sideways supporting the index finger. Middle: multiple contact areas. Thumb (and not index finger) is used to rub to and fro. It is assumed that typical movement ranges are about 20 mm. Right: various banknote properties are perceived with three fingertips (thumb, index and middle fingers). Illustrations by Wijntjes (2009)

The haptic exploration of banknotes was studied by Wijntjes (2009). This study indicated that a cash handler receiving a banknote will examine it haptically before placing it in the cash register. Usually a banknote is held between two fingers, the index finger on the reverse and the thumb on the front. The (side of the) middle finger may assist the index finger, exerting counter-pressure to the thumb. Some specific interactions are illustrated in Fig. 2. The picture on the left shows the bending of the paper, the picture in the middle shows planar movement of the thumb and the picture on the right shows the multiple contact areas. The cash handler thus perceives various banknote properties such as its structure and raised ink.

Zondervan et al. (2019) carried out an in-house study for De Nederlandsche Bank in which two “mystery shoppers” purchased a product at 30 shops and paid with an artificially modified genuine banknote. The cashier’s behavior was assessed when they were confronted with these “suspicious” banknotes. One of the findings was that approximately half of the retailers authenticate banknotes with the tips of their fingers.

Prior haptic perception research has shown that humans are very good at recognizing common objects like paper within only a few seconds on the basis of touch alone (Lederman & Klatzky, 1993). Tactile information is processed even if people do not deliberately intend to do so. According to De Heij (2017) several studies have shown that people are triggered to perform an authenticity check on a banknote that they just received when “it felt different.” In line with this, the ECB recognizes that the feel of a banknote is an important feature for detecting counterfeits. The feel includes the paper itself (“feel the banknote, it is crispy and firm”) and the raised print (“feel the short, raised lines on the left and the right edges of the banknote. The main image and the large value numeral also feel thicker”) (European Central Bank, 2013). The ink layer of the banknote is in general up to about 60 μm high. However, this height decreases when banknotes are used intensively. According to De Heij (2017) deterioration of banknotes is caused by relaxation of the paper fibers, and also by all sorts of wear and tear. Wrinkles in a banknote will create a “tactile noise level.”

A study by Raymond (2017) was designed to allow for perception testing and discrimination based on “intaglio only” (raised print). Different from the present study, the banknotes were specifically manufactured for this study. Respondents had to learn about the fantasy notes and the counterfeits were made artificially (that is, they were not removed from circulation as in the current study). Raymond et al., used three soil levels and three variants of counterfeits, similar to what, according to her, is typically seen in actual counterfeits. The results showed that sensitivity to detect counterfeit was adequate across all soil levels, even when very high-quality counterfeits were presented. Raymond concluded that tactile information affords better counterfeit detection than visual information, regardless of soil level.

Next to *intaglio*, the substrate or matter of the banknote is useful for authentication purposes. A 2013 cash survey by the Bank of Spain (Pérez, Guinea, & Negueruela, 2014) indicated that this was the most frequently verified security feature by both the general public and retailers. A study by Summers, Irwin, and Brady (2008) was conducted on the discrimination of 10 different types of plain paper on the basis of only a few seconds’ contact. Summers concluded that two perceptual dimensions, namely roughness and stiffness, are used to discriminate the paper. However, as with raised ink, a drawback of these factors is that they change dramatically over the banknote’s lifetime.

To quantify the potential of tactile discrimination in counterfeit detection, in one of our current experiments we included a condition in which participants could only feel the banknote. Comparing this condition with a “see-only” condition allowed us to quantify how important tactile information really is. Finally, the experiment also comprised a condition combining vision and touch, which is more similar to real-life transactions. As far as we know, multi-sensory authentication of banknotes has only been previously investigated by Klein, Gadbois, and Christie (2004). In subtests of this study, the objective was to compare inspection of banknotes using sight alone, touch alone and sight and touch combined. In the sight condition, the notes were put in plastic sleeves so that the participants could not feel them. In the touch condition the participants were allowed to touch the notes, but sight of the notes was blocked by a screen. Participants performed better when they saw the notes while being unable to touch them (yielding an 87% detection rate) than vice versa (74%). When sight and touch were combined the detection rate was, on average, 92%.

In sum, we wanted to know how well experts and non-experts are able to authenticate banknotes using different senses and how this authentication is affected by exposure time. We studied this in two separate experiments. In Experiment 1, the task for participants was to distinguish images of genuine banknotes from counterfeits by visual inspection on a computer display. In Experiment 2, participants had to discriminate physical genuine banknotes and counterfeits by only touching them or by touching and seeing them.

## Method for Experiment 1: “looking” (screen test)

### Participants

Participants from the general public were recruited between November 2018 and February 2019 by approaching persons at locations like community centers, schools, fairs, clubs, etc. It was explained that both test leaders work at DNB and that research was conducted to investigate how well people can detect counterfeits as this is important information for central banks. The tests were done on a voluntary basis. Every time before the test started the same introduction was read out loud by the test leader (Additional file 1). All received a USB-stick in the form of a gold bar as a small gift (unannounced and only after the test). Most people declared after the test that it was interesting and that they liked doing it. Sixty-three participants from the general public performed the screen test for all three time conditions (maximum of 500 ms, 1000 ms and 10 s). The number of male and female participants was approximately equal and the age categories were well-balanced. As such, in this respect, one can argue that our sample was adequately representing the Dutch population consistent with CBS demographic statistics (Centraal Bureau voor de Statistiek Statline, 2020).

Experts were defined as people working at a national central bank, having counterfeits as an area of expertise in their work. This means that they could be, for instance, employees who analyze intercepted counterfeits on a daily basis at the national analysis center at DNB or at another national central bank from the Eurosystem. In these analysis centers counterfeits that are removed from circulation are registered and stored. Experts could also be employees advising on policy to combat counterfeiting. Fourteen experts participated in Experiment 1.

### Stimuli

**Fig. 3:**
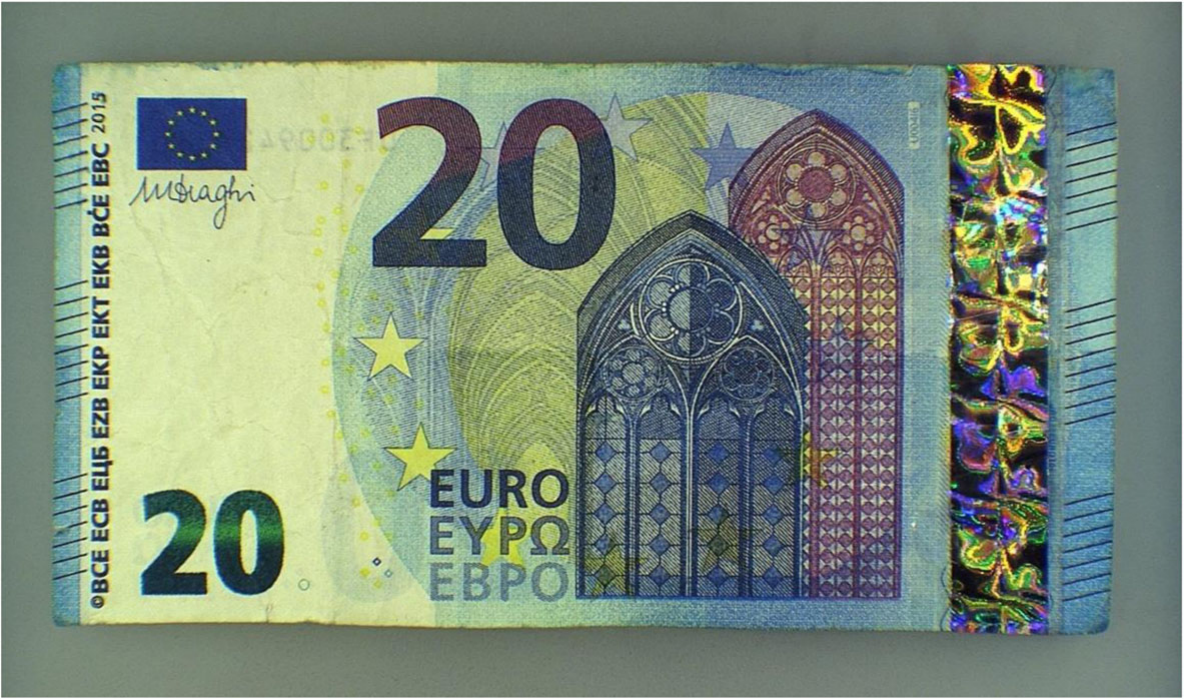
One of the counterfeits in the test set. The different foil is particularly visible. The counterfeit mimics a EUR 20 banknote from the second series

To create a test set for the present experiment we made use of counterfeits that were stored at the national analysis center of De Nederlandsche Bank. The 20 counterfeits in the test set were selected on the basis of the following criteria:

- The counterfeits were used at least once in real life (at least one person had been tricked in real life by this counterfeit). This means that they had to be taken out of circulation
- The denominations were those that tend to be counterfeited the most often (EUR 20 and EUR 50 banknotes). (European Central Bank, 2019)
- The two banknote series (ES1 and ES2) that were in circulation at the time of the tests were equally represented
- The counterfeits varied in mimicking quality. Most counterfeits were simply made with a copier, sometimes with an imitated foil attached (see, for example, Fig. 3). One of the samples was a so-called “composed note,”, i.e., partly genuine and partly counterfeit, which is considered to be a counterfeit in the Eurosystem
- The counterfeits varied in fitness quality. The counterfeited banknotes should not feel or look more worn than the genuine banknotes

Next, the test set consisted of 40 used, genuine banknotes, which were still fit for usage. The genuine banknotes were the same denominations and of the same series as the counterfeits. The proportion between genuine and counterfeit was thus 2:1. This is a much higher probability of encountering a counterfeit than in real life, which is, as noted earlier, roughly 0.003%. Nonetheless, this was necessary to obtain sufficient measurements per condition. Participants did not know exactly how many counterfeits to expect, but they were told that “most banknotes are real, but a considerable number are fake.” The reason for this phrasing was to ensure that participants could not calculate when they “were done” and then stop reporting counterfeits, as well as to let participants know that genuine banknotes are in the majority, so they would not be too easily triggered to declare a banknote as counterfeit. See Table 1 for an overview of the contents of the test set.

**Table 1:** Description of our stimulus set

		Counterfeit	Genuine	Total
EUR 20				
First series (ES1)		5	10	15
Second series (ES2)		5	10	15
EUR 50				
First series (ES1)		5	10	15
Second series (ES2)		5	10	15
**Total**		**20**	**40**	**60**

In the screen test, images of the 60 banknotes were displayed in JPG format, 2448 × 1956, resolution 300 dpi. The images were made with a Video Spectral Comparator 8000. The images were made in direct white-light conditions, so that the reverse of the note was not visible through the front, as is the case with transparent lightning. The disadvantage of this method is that “look through” elements, like the watermark and thread, are hardly visible. On the other hand, in everyday life these elements can only be seen when holding the banknote in front of a light source, which normally does not happen in cash transactions.

### Procedure

The screen on which the banknotes were presented was a MultiSync PA242W. The “auto brightness” function was enabled so that the brightness level of the screen changed automatically according to the lighting conditions of the room. The pictures were enlarged 1.5 times to better mimic real life, as 40 cm is approximately the distance from the eye to a banknote in hand and 60 cm was approximately the distance from the eye to the monitor.

**Fig. 4:**
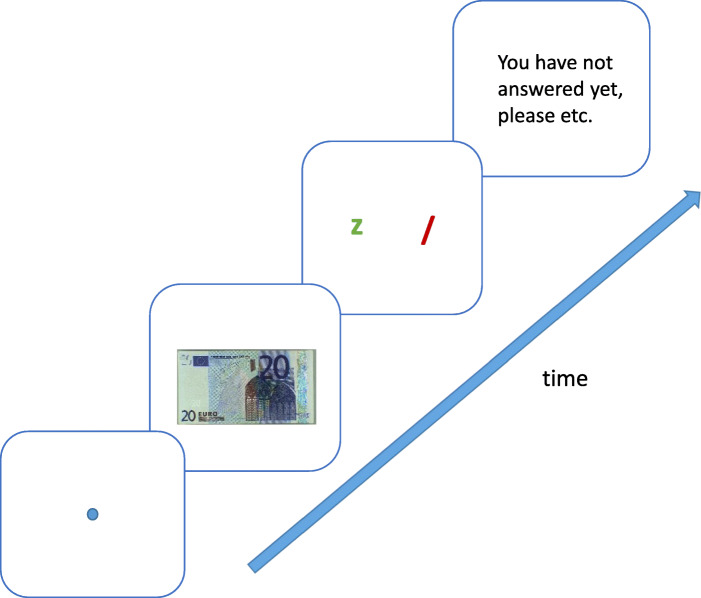
Procedure screen test. The core structure of the trial. Every trial started with a fixation dot in the center, for 500 ms, followed by a banknote (either EUR 20 or EUR 50, either genuine or counterfeit, either first or second series). The display duration was either 500 ms, 1000 ms or maximum 10 s and varied between blocks. In case of not pressing the right key a reminder was shown

Figure 4 shows the trial procedure. Every trial started with a fixation dot in the center of the screen for 500 ms followed by a picture of the front side of a banknote (with the pictures of windows and gateways). There were three exposure durations of either 500 ms, 1000 ms or 10 s (or until response) tested in separate blocks. For each participant, the order of presentation of the blocks was random. Within each block, all 60 banknotes were presented in random order. Hence, each banknote was shown three times to each participant. Following the display presentation, participants were required to press the key “z” on the keyboard if they thought that the banknote was genuine or “/” if they thought that it was a counterfeit. They could press the key the moment they wanted to answer, so they did not have to wait until stimulus offset. For the sake of convenience, the keys were marked with green sticker for the “z” key and a red one for the “/” key. Participants received six practice trials. The experiment was run on OpenSesame software (Mathôt, Schreij, & Theeuwes, 2012).

The total test time took on average 25 min. After the test the participants were invited to fill in a short questionnaire, including questions on demographics, authenticating technique and professional cash experience.

## Method for Experiment 2: “feel” and “look and feel” (physical test)

### Participants

The method of assessing public and experts was the same as it was in Experiment 1. In total 40 participants (10 experts and 30 non-experts) were tested in Experiment 2. All 10 experts performed the task both in the feel-only condition and in the look-and-feel condition, whereas the 30 non-experts were divided equally between these two conditions.

### Stimuli and procedure

The physical banknotes that were used to create images for this screen test were also used in Experiment 2. In this second experiment participants were asked to authenticate the notes one by one as they were handed over to them, but half of the participants were blindfolded so that they could only *feel* the banknotes. The physical test was to find out how participants would perform if (1) they could *only feel *the banknotes, and (2) how they perform if they can *both see and feel *the banknotes. It was intended to compare the results of this second experiment with the results of the first experiment. In the physical test, all banknotes are handled by each participant. This handling causes deterioration in banknote quality, which means that the test set was not exactly the same across the whole experiment.

Banknotes from a stack were handed over to the participants one by one by the test leaders with the front side up. The test leader did not look at the banknote in order to avoid giving clues as to whether banknotes were genuine. The banknotes could only be seen by the participants the moment that they were handed over, as the rest of the stack was kept under the table. There were four conditions (see Table 2).

**Table 2:** Four conditions of Experiment 2

	Feel	Look and feel
**Short**	1. Taking the banknote and touching it for about 1000 ms	3. Taking, seeing and touching the banknotes for about 1000 ms
**Long**	2. Taking the banknote and touching it for a maximum of 10 s	4. Taking, seeing and touching the banknote for a maximum of 10 s

Each participant was randomly assigned to either the solely feel condition or the see and feel condition. Once assigned, each participant had to judge the 60 banknotes with a short handling time, or with a long handling time. The order of these blocks was random just as the banknotes were presented in random order.

Participants had to wear a sleep-mask blocking their vision when they were tested in the feel-only conditions. In the condition “short” the test leader placed a banknote in a hand that was held open by the participant. The participant was asked to grab it with the other hand and in one movement and place it either in front of them (feel condition) or in a box in order to prevent participants from seeing the banknotes after they had made their judgement (condition feel and see). The use of both hands is intended to make sure that there was no difference in perceptual capacity between left- and right-handed participants. Furthermore, the speed of the handling was designed to come as close as possible to the short presentation times used in the screen test. In a pilot, it was estimated that this handling would last approximately 1 s. In the condition “long” the participant could use up to 10 s to explore the banknote haptically before making an assessment. The participant could decide to use their left and/or right hand and make use of different exploratory procedures. Participants showed a large variation of exploratory tactics (fondling, movement over surface, pulling from both ends, etc.). As was noted in the “Introduction,” we did not control for the type of haptic exploration employed by the participants. The handling of the banknotes was recorded on video (note that only the participants’ hands were filmed). It was made clear to the participants that their faces would not be filmed or recognized. The filming was done in order to analyze off-line the exact duration of the authentication action (from receiving the banknote until putting it down). The analysis was done with Windows Movie Maker on Windows 10.

### Performance analysis

In order to determine how well participants were able to detect counterfeits we used measures derived from Signal Detection Theory (SDT). Participants may respond to a stimulus with a simple yes or no (“yes, the banknote was fake” or “no, the banknote was not fake”). This gives the following responses. Fake banknotes could be correctly classified as counterfeit (“hit”), a fake banknote could be incorrectly classified as genuine (“miss”), a genuine banknote could be classified as counterfeit (“false alarm”) and a genuine banknote could be correctly classified as genuine (“correct rejection”).

Counterfeits are not reimbursed by central banks. So, to avoid money loss, it is key for people to recognize a counterfeit before accepting. So, a high hit rate (in the test the number of hits divided by 20 counterfeits in the test set) is crucial. However, a low false-alarm rate (the number of false alarms divided by 40 genuine banknotes in the test set), is also important for a good functioning of cash as a payment method. The ability to discriminate genuine banknotes from counterfeits is called sensitivity, combining hit and false-alarm rates. One of the most commonly used statistics for computing sensitivity is *d’*, which can be estimated by deducting the z-transformed probability of false alarms from the z-transformed probability of hits:

$$ d^{\prime }=\mathrm{z}\left(\mathrm{hit}\ \mathrm{rate}\right)-\mathrm{z}\left(\mathrm{false}-\mathrm{alarm}\ \mathrm{rate}\right). $$

A *d’* score of 0 signals an inability to distinguish counterfeits from genuine banknotes. According to Raymond (2017) a *d’* of 1.25 represents a reasonably good performance in sensitivity in banknote authentication. The maximum *d’* score that can be obtained in this study is 3.92.

Furthermore, people may have different decision-making strategies. The response bias is the extent to which one response is more probable than another. That is, a receiver may be more likely to respond that a stimulus is present (the banknote is a counterfeit) or more likely to respond that a stimulus is not present (the banknote is genuine). A commonly used statistic for this response bias is *β*. A low *β*-value indicates that a participant scored both a lot of hits and false alarms (liberal criterion) whereas a high *β* corresponds with a few hits and a low number of false alarms (conservative criterion). The bias can be estimated by calculating:

$$ \beta =\mathrm{z}\left(\mathrm{hit}\ \mathrm{rate}\right)/\mathrm{z}\left(\mathrm{false}-\mathrm{alarm}\ \mathrm{rate}\right). $$

Values of *β* larger than 1 indicate a conservative criterion. Sensitivity and bias are not defined when the hit rate and/or the false-alarm rate is zero or 1. Therefore, the maximum hit rate is set at 0.975 and the minimum false-alarm rate at 0.025.

## Results

Outliers were removed by excluding data of each participant in the screen test that had a sensitivity score in one or more of the three conditions above the mean plus 2.5 standard deviations (SD) (two participants) or below the mean minus 2.5 SD (one participant). Exclusion of the results of these three participants only affected the average sensitivity scores marginally.

Note that series (ES1, ES2) and denominations (EUR 20, EUR 50) were collapsed in all analyses.

### Results for Experiment 1 (screen test)

**Fig. 5:**
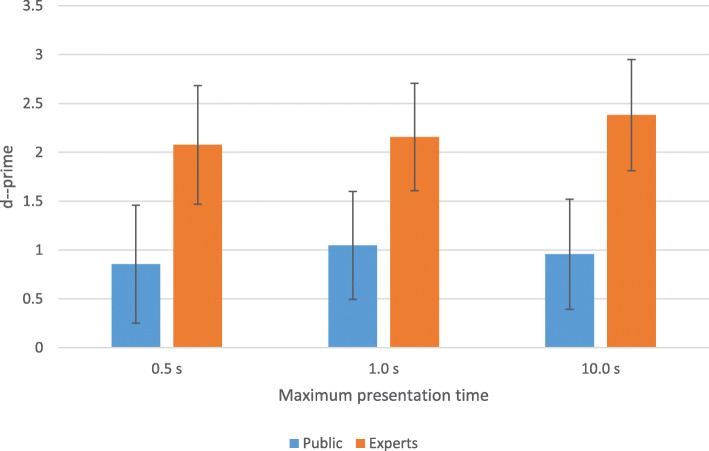
Experiment 1 mean sensitivity scores and their error bars (standard deviation (SD)) as a function of expertise and exposure time

Figure 5 presents the average sensitivity scores for Experiment 1 for all participants, and is broken down into experts and the general public. For the analysis a two-way mixed analysis of variance (ANOVA) was used: Expertise (two levels; between groups) x Time (three levels; within groups). As expected, the experts performed overall much better than participants from the general public (F(1,72) = 68.54, *p* < 0.001). There was no reliable statistical evidence that the three time conditions differed from each other (F(2,144) = 2.80, *p* = 0.0642), although there was a trend. Most participants, both experts and the general public, claimed that they were basically guessing when the banknotes were presented for only 500 ms. However, they performed in fact substantially above chance level even for the shortest display duration as the average sensitivity score by the public in the shortest display duration was 0.855 which was significantly different from chance level (zero) (one-sample *t* test: t(59) = 10.98, *p* < 0.0001). Even when taking out the counterfeit that the public recognized most often (in 88% of all displays), sensitivity was still well above chance level: t(59) = 6.63, *p* < 0.0001).

There was no evidence for an interaction effect between exposure time and level of expertise (F(2, 144) = 1.65, *p* = 0.1951).

**Fig. 6:**
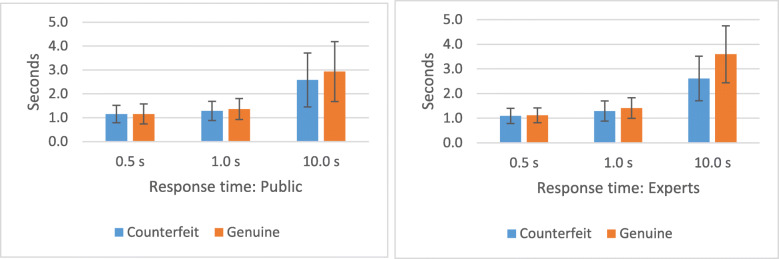
Mean response time and their error bars (standard deviation (SD)) public (left) and experts (right): screen conditions

Figure 6 shows the response times (RTs) for public and experts in Experiment 1. RT was measured as the time from the onset of the stimulus to the pressing of the key on the keyboard. We applied a 2 x 3 x 2 mixed model ANOVA on RT with level of expertise as a between-subject factor and exposure time and genuineness of the banknote (genuine or false) as within-subject factors. The response time of the general public did not differ from that of experts (F(1,75) = 0.50, *p* = 0.4806). There was a main effect for condition presentation time (F(2,150) = 249.70, *p* < 0.0001). Longer presentation time resulted in longer reaction times. Also, there was a main effect of genuineness (F(1,75) = 13.27, *p* = 0.0005). Participants took more time to decide that a banknote was genuine when it was genuine (1.930 s) than when they decided that it was counterfeited when it was in fact counterfeited (1.667 s). There was an interaction between exposure time and genuineness (*F* = 8.22, *p* = 0.0004), such that when banknotes were presented only for a short period of time, participants took the same time to authenticate counterfeits and genuine banknotes, whereas when the banknotes were presented for a longer time, participants took more time to classify genuine banknotes (t(150) = 5.39, *p* < 0.0001).

### Results for Experiment 2 (physical test)

**Fig. 7:**
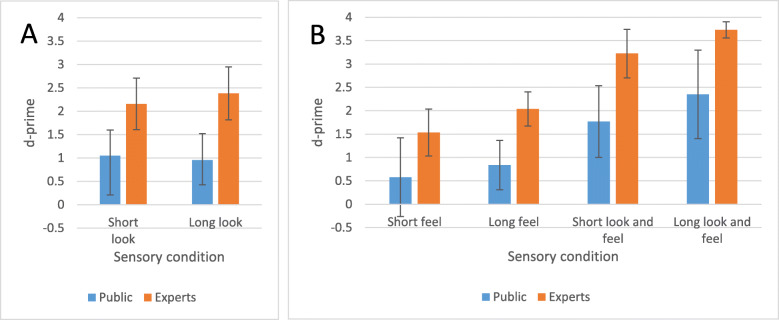
Comparing mean sensitivity scores and their error bars (standard deviation (SD)) as a function of exposure time, perceptual modality and expertise across both experiments. **a** Results for Experiment 1 (solely gauging vision). **b** Results for Experiment 2 (gauging touch and the combination of touch and vision)

Figure 7b shows the sensitivity scores for the public and the experts in the second experiment, in which physical banknotes were used. These scores were analyzed with a mixed ANOVA: Expertise (two levels; between) x Time (two levels; within) x Condition (two levels; between). All main effects were significant. Experts were again clearly better than participants from the general public, F(1,36) = 32,77, *p* < 0.0001. The combination of vision and touch led to better performance than solely touching the banknote (F(1,36) = 48.72, *p* < 0.0001). Unlike Experiment 1, when involving touch, exposure time had a significant effect (F(1,36) = 9.90, *p* = 0.0033), with longer exposure times leading to better performance. Importantly, even under the least optimal circumstances (solely touching the banknote briefly) the public (*d’ =* 0.58) scored significantly above chance level (one-sample *t* test: t(14) = 2.66, *p* = 0.0186). When taking out the data of the counterfeit that was best recognized (in 14 out of 15 times in the short feeling condition), it was borderline significantly above chance: t(14) = 2.07, *p* = 0.0576.

In the physical test the time that it took for each participant to handle each banknote was measured during the experiment and analyzed later off line. Table 3 provides the average handling times per condition. A *t* test showed that public and expert participants did not differ in handling time: t(37) = 0.42, *p* = 0.677).

**Table 3:** Average handling time per condition

Public				Experts			
Look and feel		Feel		Look and feel		Feel	
Short	Long	Short	Long	Short	Long	Short	Long
1270 ms	4580 ms	1420 ms	6500 ms	1300 ms	2370 ms	1340 ms	7990 ms

### Results of Experiments 1 and 2 combined

In this section we compared the results of Experiment 1 (vision from a computer screen) with the results of Experiment 2 (feel only and feel and look). In this analysis, for the short-exposure duration of Experiment 1 we used the 1000-ms condition, which was more or less comparable to the 1300-ms short-exposure duration of Experiment 2.The results of both tests were combined and analyzed with a three-way mixed ANOVA design, Expertise (two levels; between) x Exposure time (two levels; within) x Perceptual modality (three levels; between).

Figure 7 displays the results. All main effects are significant. As in both experiments separately, experts are better than the general public at detecting counterfeit banknotes, F(1,108) = 79,10, *p* < 0.0001. Exposure time had an effect, F(1,108) = 12,57, *p* = 0.0006 as well as the perceptual modality used F(2, 108) = 34.53, *p* < 0.0001. There is a hint towards an interaction between exposure time and perceptual modality (F(2,108) = 3.01, *p* = 0.0537) such that the effect of exposure time was greater when solely touching than when solely seeing the banknote, while combining the two senses allowed for the strongest beneficial effect of increased exposure time.

### Response biases

**Fig. 8:**
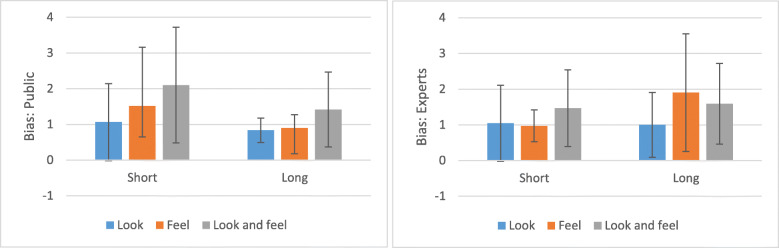
Average response biases and their error bars (standard deviation (SD)) as a function of expertise and perceptual modality

Relative to experts, the public had a different response bias, F(1,108) = 6.51, *p* = 0.0121. Participants of the general public had on average a lower *β*, meaning that they tended to declare banknotes more often as counterfeit, resulting in more hits and more false alarms. Exposure time modulated this response bias (F(1,108) = 4.35, *p* = 0.0394)). When granted only a short period of time, participants were more reluctant to judge the banknote as a counterfeit than when they had more time. Perceptual modality also impacted the bias, F(2,108) = 11.80, *p* < 0.0001. The look-and-feel condition made participants the most conservative, i.e., induced a tendency to classify a banknote as authentic. As can be seen from Fig. 8, almost all values for *β* are > 1, which means that the participants employed on average a conservative criterion.

## General discussion

This study investigated the ability of participants (experts and non-experts, i.e., general public) to authenticate euro banknotes as a function of expertise, perceptual modality (sight and touch) and exposure duration. The results suggest that when solely seeing banknotes, participants from the public did well above chance even with an exposure duration of 500 ms and even when taking out the counterfeit that was most obviously fake. As such, interestingly, they did much better than what the participants themselves expected, as most participants had the idea that they were plainly guessing. Critically, looking longer at the banknotes (1000 ms or 10 s, until response) did not improve performance. This suggests that one’s ability to detect counterfeits when solely relying on vision, is largely dependent on the first glance. In this respect a study by Raymond and Jones (2019) is relevant. This study suggested that a poor performance in authentication is especially obtained when people are not able to strategically fixate on security features that they know (for example, the hologram). Perception of scene gist (or other global properties) that do not rely on precise eye-fixation of security features are insufficient to support a satisfying banknote authentication. The results of the experts show a similar pattern regarding the necessary time as for the public: the performance of experts is much better but the performance does not increase much, if anything, with longer exposure durations. For the experts, it is as if a glimpse is enough to authenticate.

Reaction times indicated that it took longer to respond to a genuine banknote than to a counterfeit banknote. The reason that response times are longer for genuine banknotes might be that people look for evidence that the banknote is fake (a “target present” trial) and, thus, will not stop looking for such a clue until the whole banknote is scanned, thus delaying the “genuine” response (see, for example, Wolfe, 2012). Note that there was no difference in RTs between the public and experts. This particular result aligns with the response bias observed in our experiments. Participants were inclined to classify banknotes as counterfeits, which may have led them to continue looking for anomalies in the case of genuine banknotes.

Crucially, solely touching the banknote for a second yields the worst performance (Fig. 8). The conception that vision is crucial in counterfeit detection is consistent with the findings of Klein et al. (2004) who tested cashiers handling Canadian banknotes. Klein et al. (2004) showed that performance was better with notes that could be seen but not touched, than vice versa. Hence, our study and that of Klein et al. speak univocally against the common notion that only feeling a banknote is enough to instantly know that a banknote is fake. Note, however, that moving over the surface was basically not possible in the 1-s touch condition. Movement of the finger is necessary to perceive the roughness of surfaces with sandpaper particles smaller than 30 μm (Kappers & Bergmann Tiest, 2013). Hence, in future banknote design, central bankers should continue to address both senses with visual and tactile characteristics.

On average the public performed well above chance (a *d’* between 0.6 and 1.0) when solely looking or feeling, although worse than what could be called a “reasonably good performance” marked by *d’* = 1.25; a criterion introduced by Raymond (2017). Note that in the “feel and look” condition the general public scored well above this threshold of 1.25, as they had an averaged *d’* of about 1.8 in the short-exposure condition and 2.4 in the long-exposure condition.

It is not surprising that the experts performed much better than the general public. Yet, what this indicates is that with more training and instruction, the performance of the general public could be much improved. Furthermore, the result that seeing and touching the physical banknotes results in much better performance than solely seeing the images on a screen suggests that, when developing new security features, one should not only perform perception tests on a computer screen but instead have tests that also involve actual banknotes.

In closing, we are compelled to address some caveats. We tried to have the experimental conditions reflect normal handling of banknotes, but this was at best only an approximation. Seeing a banknote from a screen is different from looking at it in real life. For instance, so-called “tilt features” such as optical variable ink, cannot be detected when the note is statically displayed on a screen. Additionally, the way in which participants handled (i.e., touched) banknotes in the 1-s condition in our study might, due to task demands, have been different from how they would (more casually) handle the banknote in real life. Indeed, it is generally assumed that in daily life people implicitly check banknotes for authenticity, most likely using Type-1 processing as defined by Kahneman (2010), while rarely engaging in Type-2 processing, entailing a deliberate check of whether or not banknote is fake. In contrast, our participants were likely to do so, as they were explicitly instructed on it.

What do these considerations imply for the generalizability of our results? It may well be that the observer’s ability to view the banknote at various angles would in real life induce a beneficial effect of exposure time when solely viewing the banknote – an effect that was absent in the present study (Fig. 5). However, we see no reason to assume that the respective contribution of visual and haptic perception in real life would be much different from what we observed here. In Experiment 2, participants were indeed able to view the banknote at various angles, and this led to an increased beneficial effect of exposure time compared to when participants could only feel the banknote (Fig. 7b). It could further be argued that while participants most likely engaged in Type-2 processing (as opposed to the Type-1 processes thought to be invoked in most cash transactions), long-exposure times in real life will most probably necessitate Type-2 processing as well; (indeed, the idea of quickly and superficially checking a banknote for 10 s would be fairly paradoxical).

Nonetheless, as potential directions for future research we may outline some ways in which to tease apart the respective contributions of visual and haptic perception in a more ecologically valid manner. In the conditions combining visual and haptic perception (our Experiment 2), one may collect video-recordings from the participant’s first-person perspective. Subsequently, these recordings may be presented to participants in a separate experiment, so that they have the type of visual information that they would normally have, while the tactile information is left out. Differences in performance between the former and the latter setting could then be solely attributed to haptic perception.

In conclusion, whereas not much is gained beyond the first glance when solely relying on vision in the process of detecting counterfeit banknotes, the addition of touch allows one to accrue more evidence over time, leading to better counterfeit detection.

## Supplementary information

**Additional file 1.**

## Data Availability

The datasets used and/or analyzed during the current study are available from the corresponding author on reasonable request.
