# Supplementary material for: Finding counterfeited banknotes: the roles of vision and touch
Source: Cogn Res Princ Implic. 2020 Aug 20;5:40. doi: 10.1186/s41235-020-00236-3 (PMC7441122; doi:10.1186/s41235-020-00236-3)
Supplement: Supplementary file 1 — Additional file 1. [file 41235_2020_236_MOESM1_ESM.docx]

Annex Instruction form in Dutch

Instructions for study Instant or in hand: physical part

*To be read out by the test leader*

< introduction and welcome >

Thank you very much for participating in this test to recognize counterfeit banknotes. One of the most important tasks of De Nederlandsche Bank is to issue banknotes that are difficult to counterfeit. By participating in this test, you can help us learn more about how good Dutch people are at distinguishing between genuine and counterfeit notes.

You will soon be handed several notes one by one. Most notes are real, but a considerable number are fake. After receiving the note, your task is to put the note in the container on your right and then indicate whether you think it is real or fake.

< *for feel conditions only*: We would like to know if just feeling a note is enough to recognize whether it is genuine. That is why we ask you to put on this sleep mask for the.>

There are two conditions:

- In what we refer to as 'the long condition' you will be given a maximum of 10 seconds to assess the ticket. At the latest when the bell rings, you put the note away, but you may also put it away earlier. Then you say whether you think it is real or fake.
- In what we call 'the short condition' you will be handed the note, and you must put it in the container in one fluent movement. Then you have to say whether you think it is real or fake.

After you have assessed all the notes, we will tell you how many genuine and fake notes you correctly identified.

At the end of this survey, you will receive a short questionnaire. Your personal data cannot be traced back from this, the information will only be used for general statistics. The form with these questions will soon be given to you.

To give you some idea of what is expected, we will first do a practice session with 6 notes. The outcome of this session will not be included in the final result, it is just intended to make sure everything works and is clear.

Do you have any questions?

Ok, if everything is clear then we'll start the practice session now, good luck!
